# The effects of antenatal depression and antidepressant treatment on placental gene expression

**DOI:** 10.3389/fncel.2014.00465

**Published:** 2015-01-13

**Authors:** Jocelien D. A. Olivier, Helena Åkerud, Alkistis Skalkidou, Helena Kaihola, Inger Sundström-Poromaa

**Affiliations:** ^1^Department of Women's and Children's Health, Uppsala UniversityUppsala, Sweden; ^2^Department of Behavioral Physiology, University of GroningenGroningen, Netherlands; ^3^Department of Medicine, Centre for Gender Medicine, Karolinska InstituteStockholm, Sweden

**Keywords:** antenatal depression, antidepressants, fetal, placenta, gene expression, microarray

## Abstract

The effects of antenatal depression and antidepressant treatment during pregnancy on both mother and child are vigorously studied, but the underlying biology for these effects is largely unknown. The placenta plays a crucial role in the growth and development of the fetus. We performed a gene expression study on the fetal side of the placenta to investigate gene expression patterns in mothers with antenatal depression and in mothers using antidepressant treatment during pregnancy. Placental samples from mothers with normal pregnancies, from mothers with antenatal depression, and from mothers using antidepressants were collected. We performed a pilot microarray study to investigate alterations in the gene expression and selected several genes from the microarray for biological validation with qPCR in a larger sample. In mothers with antenatal depression 108 genes were differentially expressed, whereas 109 genes were differentially expressed in those using antidepressants. Validation of the microarray revealed more robust gene expression differences in the seven genes picked for confirmation in antidepressant-treated women than in depressed women. Among the genes that were validated *ROCK2* and *C12orf39* were differentially expressed in both depressed and antidepressant-treated women, whereas *ROCK1, GCC2, KTN1*, and *DNM1L* were only differentially expressed in the antidepressant-treated women. In conclusion, antenatal depression and antidepressant exposure during pregnancy are associated with altered gene expression in the placenta. Findings on those genes picked for validation were more robust among antidepressant-treated women than in depressed women, possibly due to the fact that depression is a multifactorial condition with varying degrees of endocrine disruption. It remains to be established whether the alterations found in the gene expression of the placenta are found in the fetus as well.

## Introduction

Unfortunately pregnancy is not a lighthearted period for all women. About 10% of pregnant women in economically developed countries and up to 25% of pregnant women in poorer countries develop symptoms of depression, such as fatigue, trouble sleeping, sense of sadness or hopelessness, during pregnancy (O'Keane and Marsh, 2007). The 5th edition of the Diagnostic and Statistical Manual of Mental Disorders (DSM-V) also acknowledged the peripartum onset of depression (American Psychiatric Association, 2013). Antenatal depression is not only affecting the mother's well-being but also affects the unborn child and has been associated with child internalizing difficulties (Barker et al., 2011), attention problems (Van Batenburg-Eddes et al., 2012), and violent behavior during adolescence (Hay et al., 2010). Moreover, the risk of developing depression during adolescence (Pawlby et al., 2009) or adulthood (Pearson et al., 2013) is higher. Although the genetic setup of the mother, the hormonal/reproductive history, current stressors, and life experiences are known risk factors (Miller and LaRusso, 2011), the underlying biological mechanisms of antenatal depression and especially its influence on the developing child remain largely unknown. So far one of the mainly suggested biological mechanism underlying the effects of antenatal depression is the activation of the HPA-axis (reviewed in: Field, 2011; Olivier et al., 2014; Waters et al., 2014). In addition to increased cortisol levels Field et al. (2004) also reported on reduced serotonin and dopamine levels in urine samples of depressed pregnant women. Alterations in cortisol/HPA axis responses or in catecholamines/serotonin may explain some effects in the offspring, however the intrauterine environment is directly passed into the embryo-fetal epigenetic programming. For this reason exposure to antenatal depression *in utero* may also increase the risk for adverse outcome in the offspring via epigenetic alterations (Babenko et al., 2015). Several treatments for antenatal depression are available, including antidepressant treatment. Antidepressants pass the placenta and are found in the amniotic fluid (Hostetter et al., 2000; Loughhead et al., 2006). Although the exact effect on the offspring is unknown, the use of antidepressants during pregnancy has increased during the last decades. From 1998 to 2005 a 300% increase in antidepressant use during pregnancy was reported (Alwan et al., 2011) and this number is still increasing. Selective serotonin reuptake inhibitors (SSRIs) are the most frequently used antidepressants during pregnancy (Andrade et al., 2008), and are generally considered safe (Gentile, 2005). However, epidemiologic studies have found associations between SSRI use and neurodevelopmental disorders, e.g., autism (Croen et al., 2011; Rai et al., 2013), and attention-deficit hyperactivity disorder (Clements et al., 2014). Cohort studies are underway for the study of SSRI use during pregnancy and the neurodevelopmental disorders in the offspring (Malm et al., 2012). Although some SSRI effects during pregnancy have been reported in the offspring (see: Olivier et al., 2013; Bourke et al., 2014) there is still a great need to investigate the molecular mechanisms involved in antenatal depression that may be altered by antidepressants. By unraveling these pathways we generate more insight into the effects of antenatal depression and antidepressant treatment on the developing child, which ultimately helps in future decisions of using antidepressants during pregnancy.

The placenta plays a pivotal role in supporting fetal growth and development and is a crucial regulator of maternal-fetal interactions and fetal brain development (Hsiao and Patterson, 2012). In fact, placental serotonin synthesis directly modulate fetal brain development (Bonnin et al., 2011). As the placenta carries important information about the pregnancy, investigation of the placenta provides valuable insight to the molecular mechanisms that may have both immediate and long lasting effects on fetal health. This study was designed as a hypothesis-generating study and investigated the impact of antenatal depression and antidepressant treatment during pregnancy on the gene expression in the fetal placenta. Placental samples from mothers with normal pregnancies, from mothers with antenatal depression, and from mothers using antidepressants were collected from the “Biology, Affect, Stress, Imaging and Cognition in Pregnancy and the Puerperium” (BASIC) project. In a first pilot microarray experiment 108 genes were **differentially** expressed in antenatal depressed mothers whereas 109 genes were **differentially** expressed in those using antidepressants. Of these genes, seven were chosen for biological validation in a larger sample.

## Materials and methods

### Subjects

This study was carried out at the Department of Women's and Children's health, Uppsala University Hospital, as part of an ongoing longitudinal study on antenatal and postpartum depression: the Biology, Affect, Stress, Imaging and Cognition in Pregnancy and the Puerperium (BASIC) project. The BASIC project started in 2010 and aims to include a study population of 5000 pregnant women in the Uppsala County. Women attending the routine ultrasound examination (gestational week 16–17) at Uppsala University Hospital are approached for participation, enabling a population-based sampling. Upon informed consent, women fill out web-based questionnaires in gestational week 17 and 32 including questions on physical and socio-demographic characteristics, medical, psychiatric, gynecologic and obstetric history variables, lifestyle, medication parameters, and the Swedish version of the Edinburgh Postnatal Depression Scale (EPDS). Information concerning the maternal depression, SSRI use, delivery and neonatal outcome were retrieved from the medical records. Placental biopsies are collected at delivery.

For the specific aim of this sub-study, inclusion criteria were women of Caucasian origin, normal pregnancies and deliveries and healthy offspring (no diagnoses and no admittance to neonatal care). Exclusion criteria were smoking or alcohol use during pregnancy, any daily use of prescribed drugs during pregnancy, any other chronic condition or disease, gestational age <35 weeks, and maternal age <18 or >42 years. Women on antidepressants used their treatment during the entire pregnancy in clinically relevant doses (low-dose use was excluded). The study was approved by the Regional Ethics Committee, Uppsala, Sweden, and performed in accordance with relevant guidelines and regulations.

#### Study population for micro-array analysis

Women with pregnancies complicated by ongoing depression (*n* = 5), SSRI treatment (*n* = 5) and women with normal pregnancies (*n* = 10) were selected from the BASIC biobank. Depressed women had medical records confirming major depression and ongoing treatment for their depression in terms of psychotherapy. In the SSRI group sertraline (*n* = 3), fluoxetine (*n* = 1) and escitalopram (*n* = 1) was used. Women on SSRIs displayed significantly lower depression scores than depressed women, i.e., the two groups were not readily comparable (Table 1). Hence, the exposure of ongoing depression or SSRI treatment, respectively, were compared against two control groups. Controls were matched with respective depressed or SSRI-treated women by age (±2 years), BMI (±one unit) and gestational length (±1 week) on an individual level. The control group consisted of women with no history and no current symptoms/diagnoses of mood or anxiety disorders, and their EPDS scores at gestational week 17 and 32 were 6 or lower.

**Table 1:** **Microarray demographic variables in the depressed group, SSRI group, and healthy controls**.

	**Depressed Women (*n* = 5)**	**Healthy controls (*n* = 5)**	**SSRI-treated Women (*n* = 5)**	**Healthy controls (*n* = 5)**
Age (years)	31.4 ± 2.2	31.2 ± 2.4	29.2 ± 3.4	29.0 ± 3.0
Parity (*n*, median, range)	1 (0-2)	0 (0-1)	0 (0-2)	0 (0-2)
BMI (kg/m2)	22.8 ± 3.0	23.5 ± 2.5	26.9 ± 5.8	24.5 ± 4.1
Birth weight (gram)	3542 ± 461	3632 ± 342	3538 ± 225	3556 ± 275
Gender offspring (% boy)	60	40	20	80
Gestational length	276 ± 11	283 ± 5	275 ± 3	277 ± 5
EPDS score week 17	14.8 ± 7.3^a^	2.6 ± 1.8	8.0 ± 3.2	3.4 ± 1.6
EPDS score week 32	18.8 ± 5.3^b^	3.2 ± 1.6	6.3 ± 3.4	4.0 ± 1.2

*Data presented as mean ± SD or median (range)*.

a *significantly greater than both control groups, P < 0.01, ANOVA post hoc Bonferroni*.

b *significantly greater than all other groups, P < 0.001, ANOVA post hoc Bonferroni*.

#### Study population for validation of the microarray

The samples described above were extended to 24 women with pregnancies complicated by ongoing depression, 29 antidepressant-treated women and 31 women with normal pregnancies. The depressed group included the five microarray cases and 18 women with EPDS >12 in gestational week 17 *and* 32, or an EPDS score >14 on at least one time point (*n* = 1). The average EPDS score for all depressed women was >15 in gestational week 17 and 32. The EPDS questionnaire is validated for use in both pregnant and postpartum women (Gibson et al., 2009), and has been validated for the Swedish setting (Rubertsson et al., 2011). The EPDS contains ten items (rated on a scale from 0 to 3), based on the past 7 days. While its sensitivity is relatively low, a cut-off score of >12 points during pregnancy has a specificity of 98–99% for major depression (Bergvink et al., 2011). Thirteen of the depressed women were also evaluated by Mini International Neuropsychiatric Interview, which confirmed that all but one (she had social phobia) had major depressive disorder during pregnancy. In the antidepressant-treated group, women used sertraline (*n* = 11), fluoxetine (*n* = 8), citalopram/escitalopram (*n* = 7), venlafaxine (*n* = 2) and clomipramine (*n* = 1). Treatment had been initiated by primary care physicians as well as by psychiatrists.

### Sample collection, processing, and storing

Placental tissues (containing both maternal and fetal side) were obtained after delivery, rinsed carefully in sterile phosphate-buffered saline to wash off maternal and fetal blood, and frozen on dry ice within 60 min of delivery and stored at −70°C until further use. Each placenta was individually processed as a single biological replicate in the microarray and validation study.

### RNA isolation

#### Microarray study

A biopsy was taken with a 3 mm cube from the fetal side of the placenta. Total RNA was isolated using miRNeasy mini kit (Qiagen, Hilden, Germany). Tissue was lysed with QIAzol reagent (Qiagen) using a rotor-stator homogenizer (up to 33.000 rpm; Ingenieursbűro CAT M Zipper Gmbh, type x120, Staufen, Germany) and chloroform (Sigma Aldrich, St. Louis, MO, USA) was added for phase-separation. The rest of the procedure was performed as described in manufactures protocol.

#### Validation study

A biopsy was taken from the fetal side of the placenta with a 3 mm cube. Total RNA was isolated using RNeasy mini (Qiagen, Hilden, Germany). Tissue was lysed with QIAzol reagent (Qiagen) using TissueLyser (20Hz, 2 × 5 min) with stainless steel beads (Qiagen) and chloroform was added for phase-separation. The rest of the procedure was performed as described in manufactures protocol.

For both studies RNA concentration was measured with ND-1000 spectrophotometer (NanoDrop Technologies, Wilmington, Delaware, USA) and RNA quality was evaluated using the Agilent 2100 Bioanalyzer system (Agilent Technologies Inc, Palo Alto, California, USA).

### Microarray expression analysis

250 nanograms of total RNA from each sample were used to generate amplified and biotinylated sense-strand cDNA from the entire expressed genome according to the Ambion WT Expression Kit (P/N 4425209 Rev B 05/2009) and Affymetrix GeneChip® WT Terminal Labeling and Hybridization User Manual (P/N 702808 Rev. 4, Affymetrix Inc., Santa Clara, CA). GeneChip® ST Arrays (GeneChip® XXX Gene 1.0 ST Array) were hybridized for 16 h in a 45°C incubator, rotated at 60 rpm. According to the GeneChip® Expression Wash, Stain and Scan Manual (PN 702731 Rev 3, Affymetrix Inc., Santa Clara, CA) the arrays were then washed and stained using the Fluidics Station 450 and finally scanned using the GeneChip® Scanner 3000 7G.

### Microarray data analysis

The raw data was normalized in the free software Expression Console provided by Affymetrix (http://www.affymetrix.com) using the robust multi-array average (RMA) method first suggested by Li and Wong (2001) and Irizarry et al. (2003). Subsequent analysis of gene expression data was carried out in the freely available statistical computing language R (http://www.r-project.org) using packages available from the Bioconductor project (www.bioconductor.org). In order to search for differentially expressed genes between the depressed and controls, and the SSRI and the control groups an empirical Bayes moderated *t*-test with robust regression was applied (Smyth, 2004), using the “limma” package (Smyth, 2005). To address the problem of multiple testing, *p*-values were adjusted according to Benjamini and Hochberg (1995). The Genesis software, version 1.7.1 (http://genome.tugraz.at/), was used to produce hierarchical clustering and to visualize differentially expressed genes by heat maps (Sturn et al., 2002). The expression data were further analyzed using ingenuity pathway analysis (IPA) in order to determine significantly deregulated genes and pathways (Ingenuity® Systems, Mountain View, CA, USA; www.ingenuity.com). IPA reveals Top genes, which are genes with the largest normalized enrichment scores, and IPA computes a score for each network according to the fit of that network to the user-defined set of Focus Genes. The score, derived from a *p*-value, indicates the likelihood of the association between the set of focus genes (Bonferroni-corrected significance threshold of *P* < 0.05 and a fold change of 0.5) and a given pathway. The smaller the *p*-value, the less likely that the association is found due to random chance. In general *P*-values below 0.05 indicate a non-random significant association. The *p*-value is calculated using the right-tailed Fisher Exact Test (for more details see www.ingenuity.com).

### cDNA synthesis

cDNA was synthesized using SuperScriptIII reversed transcriptase (Invitrogen, Carlsbad, California, USA) according to manufacturer's protocol. Briefly, 250 ng of total RNA was used to reverse transcribe using the random primer to prepare 20 μl of cDNA.

### Real-time quantitative reverse transcriptase polymerase chain reaction analysis

The validity of the microarray results was tested via quantitative real-time PCR (qRT-PCR) employing the StepOne Plus qPCR machine (Applied Biosystems, Life Technologies, Carlsbad, California, USA). For validation we selected seven genes [*NEXN* (Hs00332124_m1), *GCC2* (Hs00206083_m1), *ROCK 1* (Hs01127699_m1), *ROCK2* (Hs00178154_m1), *DNM1* (Hs00247147_m1), *KTN1* (Hs00192160_m1), *and C12ORF39* (Hs00228976_m1)] which showed a fold change (FC) > 0.5 in the microarray. *GAPDH* (Hs99999905_m1) and *β-actin* (4326315E) were selected as reference genes for normalization. cDNA of the samples was used for quantification. TaqMan Gene expression Assay primers, probes and gene expression master mix (all Applied Biosystems, Life Technologies, Carlsbad, CA, USA) were used to run the qRT-PCR according to manufacturer's instructions. Mean plate efficiencies were calculated by LinReg.

### qPCR data analysis

All samples were performed in triplicates and averaged for further calculations. Mean normalized expression (MNE) based on the ratio between *Ct*-values of target and reference genes and the efficiency of the PCR reactions, was calculated as a measure of target gene transcription, as described previously (Muller et al., 2002; Helmestam et al., 2012). Data are presented as log_2_ MNE to illustrate the difference between the groups.

### Statistics

Clinical characteristics of women in the microarray study and the validation study were compared by means of One-Way ANOVA, followed by a Bonferroni *post hoc* test when appropriate. Differences in expression between subsets in the validation study (qPCR) were calculated using a univariate ANOVA with age, BMI, parity, and week of delivery as covariates. Data were analyzed using the SPSS 20.0 software. Level of significance was set at *P* < 0.05. Data are presented as mean ± S.E.M.

## Results

### Subjects

The demographic characteristics of the women in the microarray and validation studies are presented in Tables 1, 2. *Microarray study:* A significant group difference was found for EPDS scores at gestational week 17 [*F*_(3, 18)_ = 8.9, *P* < 0.01] and gestational week 32 [*F*_(3, 17)_ = 22.8, *P* < 0.001]. Depressed women had significantly higher EPDS scores in gestational week 17 and 32 compared with controls (*P* < 0.01), whereas women on SSRIs did not differ from controls at any time-point. In gestational week 32, depressed women also had significantly higher EPDS scores than SSRI-treated women, Table 1. *Validation study:* All women on SSRI treatment reported previous anxiety and/or depression at the first antenatal booking, whereas 41% of depressed cases had no previous psychiatric history. Women using antidepressants had a significantly shorter gestational length than controls (*P* < 0.01). As expected, significant group differences were found in EPDS scores at gestational weeks 17 [*F*_(2, 74)_ = 69.6, *P* < 0.001] and 32 [*F*_(2, 74)_ = 74.3, *P* < 0.001]. In gestational week 17 and 32, depressed women had significantly higher EPDS scores than antidepressant-treated women (*P* < 0.001 and *P* < 0.001, respectively), whom in turn had higher EPDS scores than controls (*P* < 0.001 and *P* < 0.001, respectively), Table 2. No other parameters differed between groups.

**Table 2:** **Validation demographic variables in the depressed group, SSRI group, and healthy controls**.

	**Healthy controls (*n* = 31)**	**Depressed women (*n* = 24)**	**SSRI-treated Women (*n* = 29)**
Age (years)	31.4 ± 3.9	31.1 ± 4.3	31.2 ± 4.1
Parity (*n*, median, range)	0 (0-3)	1 (0-2)	1 (0-3)
BMI (kg/m^2^)	26.0 ± 4.9	24.0 ± 6.2	27.2 ± 4.9
Systolic blood pressure in first trimester, mmHg	119 ± 13	111 ± 12	118 ± 12
Diastolic blood pressure in first trimester, mmHg	73 ± 9	69 ± 7	70 ± 7
Systolic blood pressure at last visit, mmHg	125 ± 11	119 ± 11	125 ± 10
Diastolic blood pressure at first visit, mmHg	79 ± 8	76 ± 6	77 ± 7
Lowest hemoglobin level during pregnancy, g/dl	11.7 ± 0.8	11.4 ± 0.8	11.0 ± 0.9^c^
Birth weight (gram)	3577 ± 351	3546 ± 499	3589 ± 400
Gender offspring (% boy)	65	54	45
Gestational length	281 ± 7	275 ± 11	273 ± 8^c^
EPDS score week 17	2.9 ± 1.8	15.0 ± 4.2^a^	7.7 ± 4.9^b^
EPDS score week 32	2.9 ± 1.8	15.9 ± 3.6^a^	8.7 ± 5.3^b^^d^

*Data presented as mean ± SD or median (range)*.

a *significantly higher than all other groups, P < 0.001, ANOVA post hoc Bonferroni*.

b *significantly higher than healthy control group, P < 0.001, ANOVA post hoc Bonferroni*.

c *significantly lower than healthy control group, P < 0.01, ANOVA post hoc Bonferroni*.

d *significantly lower than depressed group, P < 0.001, ANOVA post hoc Bonferroni*.

### Differentially expressed genes and pathways between the depressed and control placentas

**Figure 1:**
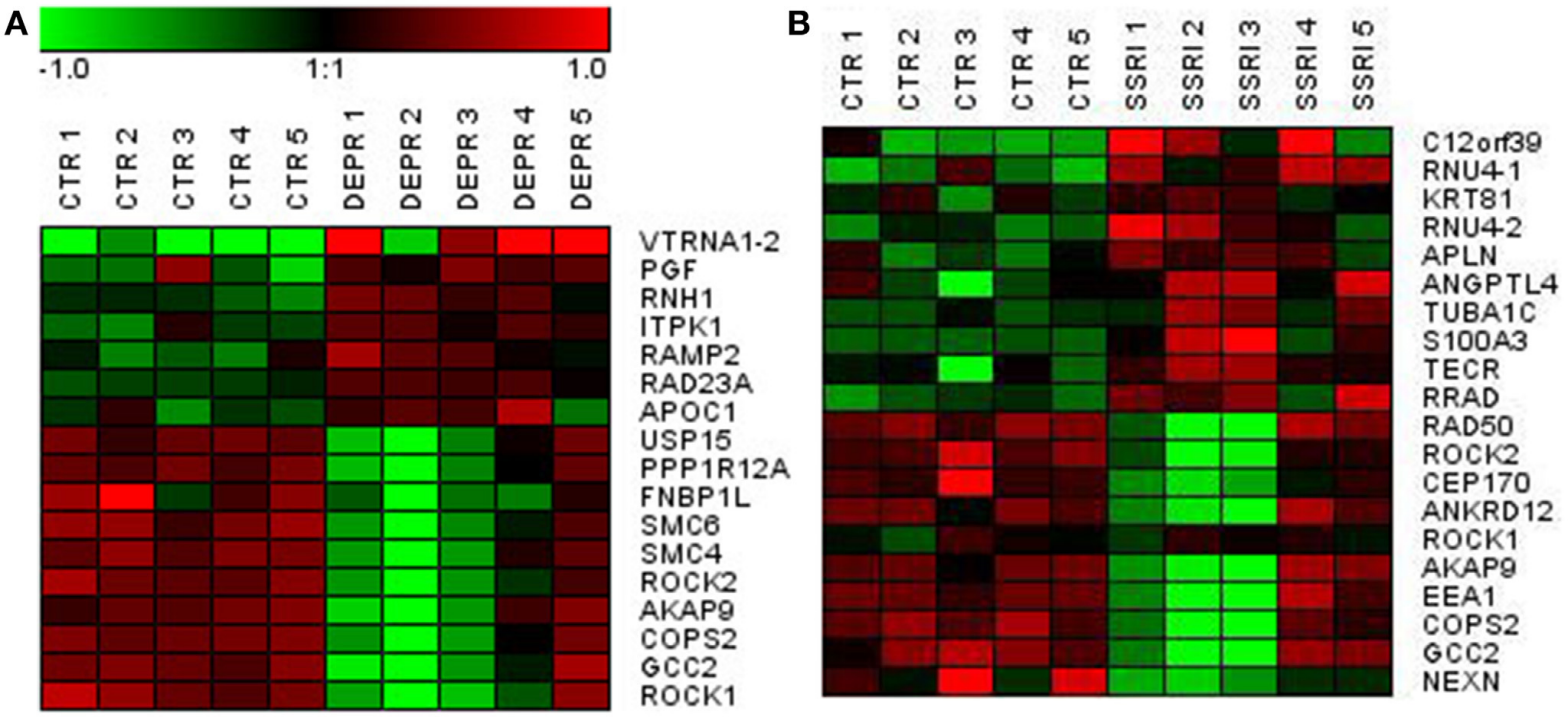
**Visualization of differentially expressed genes using hierarchical clustering of genes in depressed (A) and SSRI-treated (B) vs. control fetal placentas**.

At a Bonferroni-corrected significance threshold (*P* < 0.05) and a log2-fold change of 0.5 or higher we found 108 genes differentially expressed between the depressed women and their respective controls; 100 were down-regulated and 8 up-regulated, see Table 3. The raw microarray data is found as an excel file in the Supplementary Data. The ingenuity pathway analysis (IPA) revealed 17 differentially expressed top genes; 7 were up-regulated and 10 down-regulated. Top up- and down-regulated molecules are summarized in Table 4. We then clustered placentas according to their gene expression profiles for the 17 genes that displayed differential expression (see Figure 1A). In order to determine the biological relevance, analysis with IPA was performed, focusing on genes that differed in expression between placentas from depressed women and controls. As shown in Table 5, we identified five gene networks that were significantly enriched, classified as follows: (I) DNA Replication, Recombination, and Repair, Cellular Assembly and Organization, Cell Cycle with an IPA score of 27; (II) Cell Cycle, Cancer, Connective Tissue Disorders with a IPA score of 18; (III) Cellular movement, Hematological System Development and Function, Immune Cell Trafficking with an IPA score of 16; (IV) Cardiovascular System Development and Function, Organismal Development, Visual System Development and Function with an IPA score of 14; and (V) Molecular Transport, RNA Trafficking, Connective Tissue Disorders with an IPA score of 9 (Table 5). Further, the significant canonical pathways identified by IPA (*P* < 0.05) are shown in Table 6, along with the included genes and *p*-values. Pathways included Actin Nucleation by ARP-WASP Complex, RhoA Signaling, VEGF Signaling, Protein Kinase A Signaling, and 1D-myo-inositol Hexakisphosphate Biosynthesis V [from Ins(1,3,4)P3].

**Table 3:** **Significantly up and down regulated genes in the control vs. depressed groups (− = reduction in expression levels)**.

**Gene symbol**	**Gene name**	**Probe ID**	**Log2 fold change**
*VTRNA1-2*	vault RNA 1-2	8108629	2.280673826
*PGF*	placental growth factor	7980233	0.695475147
*RNH1 /// FLJ23519*	ribonuclease/angiogenin inhibitor 1 /// hypothetical protein FLJ23519	7945420	0.565671098
*ITPK1*	inositol 1,3,4-triphosphate 5/6 kinase	7980970	0.549075065
*mir503*	−	8175261	0.519417648
*RAD23A*	RAD23 homolog A (*S. cerevisiae*)	8026122	0.51907744
*FAM183B*	acyloxyacyl hydrolase (neutrophil)	8139160	0.50922813
*APOC1*	apolipoprotein C-I	8029536	0.502399918
*KIR3DL2 /// KIR2DS2 /// KIR2DL2 /// KIR2DL1 /// KIR2DS4 /// KIR2DL3 /// LOC727787 /// KIR2DS5*	killer cell immunoglobulin-like receptor, three domains, long cytoplasmic tail, 2 /// killer cell immunoglobulin-like receptor, two domains, short cytoplasmic tail, 2 /// killer cell immunoglobulin-like receptor, two domains, long cytoplasmic tail, 2 /// killer cell immunoglobulin-like receptor, two domains, long cytoplasmic tail, 1 /// killer cell immunoglobulin-like receptor, two domains, short cytoplasmic tail, 4 /// killer cell immunoglobulin-like receptor, two domains, long cytoplasmic tail, 3 /// similar to killer cell immunoglobulin-like receptor 3DL2 precursor (MHC class I NK cell receptor) (Natural killer-associated transcript 4) (NKAT-4) (p70 natural killer cell receptor clone CL-5) (CD158k antigen) /// killer cell immunoglobulin-like receptor, two domains, short cytoplasmic tail, 5	8031293	−0.506084964
*SNX4*	sorting nexin 4	8090256	−0.506251931
*NEK1*	NIMA (never in mitosis gene a)-related kinase 1	8103646	−0.50748106
*RALGPS2*	Ral GEF with PH domain and SH3 binding motif 2	7907657	−0.508099708
*RNF160*	ring finger protein 160	8069711	−0.50811689
−	−	8104012	−0.508994997
*RIF1*	RAP1 interacting factor homolog (yeast)	8045697	−0.511151224
*LRP2*	low density lipoprotein-related protein 2	8056611	−0.513852689
*PTBP2*	polypyrimidine tract binding protein 2	7903188	−0.517797539
*ZMAT1*	zinc finger, matrin type 1	8174119	−0.519592242
*RUFY2*	RUN and FYVE domain containing 2	7933999	−0.524450233
*SNAPC1*	small nuclear RNA activating complex, polypeptide 1, 43kDa	7974870	−0.52596952
*RNF19A*	ring finger protein 19A	8152041	−0.526515918
*SKIV2L2*	superkiller viralicidic activity 2-like 2 (*S. cerevisiae*)	8105353	−0.530773126
*ANAPC4*	anaphase promoting complex subunit 4	8094408	−0.533126207
−	−	7916667	−0.533554256
*NCKAP1*	NCK-associated protein 1	8057517	−0.53822831
*DGKH*	diacylglycerol kinase, eta	7968800	−0.538998035
*MARCH7*	membrane-associated ring finger (C3HC4) 7	8045919	−0.550742909
*HOMER1*	homer homolog 1 (Drosophila)	8112841	−0.551657356
*PHF20L1*	PHD finger protein 20-like 1	8148358	−0.551986952
*YEATS4*	YEATS domain containing 4	7957032	−0.55366204
*CHRM3*	cholinergic receptor, muscarinic 3	7910915	−0.554183212
*PDE3B*	phosphodiesterase 3B, cGMP-inhibited	7938629	−0.557038965
*BRMS1L*	breast cancer metastasis-suppressor 1-like	7973948	−0.55836529
*SNORD30*	small nucleolar RNA, C/D box 30	7948900	−0.560928764
*ZNF84*	zinc finger protein 84	7960143	−0.562597276
*MND1*	meiotic nuclear divisions 1 homolog (*S. cerevisiae*)	8097857	−0.5650204
*LOC221442*	adenylate cyclase 10 pseudogene	8119423	−0.565773386
*UBA6*	ubiquitin-like modifier activating enzyme 6	8100615	−0.566611488
*N4BP2L2*	NEDD4 binding protein 2-like 2	7970907	−0.570303348
*ZRANB2*	zinc finger, RAN-binding domain containing 2	7916969	−0.573773594
*EIF5B*	eukaryotic translation initiation factor 5B	8043861	−0.574408988
*NAP1L1*	nucleosome assembly protein 1-like 1	7965048	−0.581711065
*ZNF146*	zinc finger protein 146	8028186	−0.584609444
*BRWD3*	bromodomain and WD repeat domain containing 3	8173766	−0.592331241
*KIAA1109*	KIAA1109	8097148	−0.602191356
*SENP7*	SUMO1/sentrin specific peptidase 7	8089203	−0.603487152
*FZD6*	frizzled homolog 6 (Drosophila)	8147766	−0.605426406
*CHD9*	chromodomain helicase DNA binding protein 9	7995583	−0.607612535
*KIAA1430*	KIAA1430	8103979	−0.617503882
*RNF217*	ring finger protein 217	8121825	−0.620970732
*PCMTD1*	protein-L-isoaspartate (D-aspartate) O-methyltransferase domain containing 1	8150714	−0.635640315
*NKTR*	natural killer-tumor recognition sequence	8079079	−0.63772807
*KIF23*	kinesin family member 23	7984540	−0.649229354
*PRPF40A*	PRP40 pre-mRNA processing factor 40 homolog A (*S. cerevisiae*)	8055913	−0.652162751
−	−	8098287	−0.658215787
*ARID4A*	AT rich interactive domain 4A (RBP1-like)	7974621	−0.661691075
*LUC7L3*	LUC7-like 3 (*S. cerevisiae*)	8008493	−0.663829187
*NIPBL*	Nipped-B homolog (Drosophila)	8104944	−0.6642255
*CENPE*	centromere protein E, 312kDa	8102076	−0.664227054
*BOD1L*	biorientation of chromosomes in cell division 1-like	8099410	−0.665905703
*TAF1D*	TATA box binding protein (TBP)-associated factor, RNA polymerase I, D, 41kDa	7951008	−0.668975157
*PIBF1*	progesterone immunomodulatory binding factor 1	7969390	−0.670731182
*SENP6*	SUMO1/sentrin specific peptidase 6	8120758	−0.675080818
*SMC2*	structural maintenance of chromosomes 2	8156982	−0.681747538
*DNAJC10*	DnaJ (Hsp40) homolog, subfamily C, member 10	8046759	−0.690820266
*ZNF638*	zinc finger protein 638	8042601	−0.696757481
*STXBP3*	syntaxin binding protein 3	7903541	−0.698824428
*TTK*	TTK protein kinase	8120838	−0.699258503
*SCYL2*	SCY1-like 2 (*S. cerevisiae*)	7957806	−0.710364273
*ERBB2IP*	erbb2 interacting protein	8105681	−0.71178458
*CHD1*	chromodomain helicase DNA binding protein 1	8113305	−0.71533841
*MPP6*	membrane protein, palmitoylated 6 (MAGUK p55 subfamily member 6)	8131927	−0.715467135
*C1orf27*	chromosome 1 open reading frame 27	7908330	−0.715867564
*DNM1L*	dynamin 1-like	7954752	−0.716232722
*CEP152*	centrosomal protein 152kDa	7988537	−0.717688618
*OTUD6B*	OTU domain containing 6B	8147262	−0.735794509
*ATRX*	alpha thalassemia/mental retardation syndrome X-linked (RAD54 homolog, *S. cerevisiae*)	8173673	−0.740293061
*ZNF100*	zinc finger protein 100	8035808	−0.741413733
*KIF18A*	kinesin family member 18A	7947248	−0.743358618
*DEK*	DEK oncogene	8124144	−0.744034441
−	−	8083445	−0.766235985
−	−	8119580	−0.77576534
*SDCCAG1*	serologically defined colon cancer antigen 1	7978866	−0.776418378
*ANKRD36B*	ankyrin repeat domain 36B	8054064	−0.777582301
*ANKRD26*	ankyrin repeat domain 26	7932637	−0.779453671
*LYSMD3*	LysM, putative peptidoglycan-binding, domain containing 3	8113064	−0.780972234
*CTAGE4 /// CTAGE6 /// LOC100142659 /// LOC441294 /// hCG_2030429*	CTAGE family, member 4 /// CTAGE family, member 6 /// CTAGE family member /// similar to CTAGE6 /// CTAGE family, member 4-like	8136979	−0.78257421
*SUCLA2*	succinate-CoA ligase, ADP-forming, beta subunit	7971541	−0.78881152
*SMC5*	structural maintenance of chromosomes 5	8155770	−0.795143951
*POLK*	polymerase (DNA directed) kappa	8106303	−0.814486061
*ERGIC2*	ERGIC and golgi 2	7962013	−0.820896474
*RAD50*	RAD50 homolog (*S. cerevisiae*)	8107942	−0.827079801
*THOC2*	THO complex 2	8174893	−0.836189906
*KTN1*	kinectin 1 (kinesin receptor)	7974483	−0.866627304
*JMJD1C*	jumonji domain containing 1C	7933877	−0.86686842
*USP15*	ubiquitin specific peptidase 15	7956670	−0.869012602
*PPP1R12A*	protein phosphatase 1, regulatory (inhibitor) subunit 12A	7965123	−0.883024382
*FNBP1L*	formin binding protein 1-like	7903092	−0.886872231
*SMC6*	structural maintenance of chromosomes 6	8050443	−0.915161746
*SMC4*	structural maintenance of chromosomes 4	8083709	−0.920861102
*ROCK2*	Rho-associated, coiled-coil containing protein kinase 2	8050302	−0.924567683
*AKAP9*	A kinase (PRKA) anchor protein (yotiao) 9	8134122	−0.935693684
*ZNF252*	zinc finger protein 252	8153935	−0.939582046
*COPS2*	COP9 constitutive photomorphogenic homolog subunit 2 (Arabidopsis)	7988605	−0.975928968
*GCC2*	GRIP and coiled-coil domain containing 2	8044236	−1.024026926
*CTAGE4 /// CTAGE6 /// LOC100142659 /// LOC441294 /// hCG_2030429*	CTAGE family, member 4 /// CTAGE family, member 6 /// CTAGE family member /// similar to CTAGE6 /// CTAGE family, member 4-like	8129560	−1.025580319
*ROCK1*	Rho-associated, coiled-coil containing protein kinase 1	8022441	−1.074633032
*FLJ45950*	FLJ45950 protein	7952673	−1.131057985

**Table 4:** **Significantly up- and down-regulated top molecules in the control vs. the depressed group (− = reduction in expression levels)**.

**Gene symbol**	**Gene title**	**log2 fold change**	***P*-value**
*VTRNA1-2*	vault RNA 1-2	2.28	0.026
*PGF*	Placenta growth factor	0.70	0.013
*RNH1*	ribonuclease/angiogenin inhibitor 1	0.57	0.036
*ITPK1*	inositol 1,3,4-triphosphate 5/6 kinase	0.55	0.037
*Mir-503*	microRNA 503	0.52	0.047
*RAD23A*	RAD23 homolog A (*S. cerevisiae*)	0.52	0.013
*APOC1*	apolipoprotein C-I	0.50	0.037
*USP15*	ubiquitin specific peptidase 15	−0.87	0.026
*PPP1R12A*	protein phosphatase 1. regulatory (inhibitor) subunit 12A	−0.88	0.021
*FNBP1L*	formin binding protein 1-like	−0.89	0.046
*SMC6*	structural maintenance of chromosomes 6	−0.92	0.026
*SMC4*	structural maintenance of chromosomes 4	−0.92	0.024
*ROCK2*	Rho-associated. coiled-coil containing protein kinase 2	−0.92	0.021
*AKAP9*	A kinase (PRKA) anchor protein (yotiao) 9	−0.94	0.028
*COPS2*	COP9 constitutive photomorphogenic homolog subunit 2 (Arabidopsis)	−0.98	0.008
*GCC2*	GRIP and coiled-coil domain containing 2	−1.02	0.019
*ROCK1*	Rho-associated. coiled-coil containing protein kinase 1	−1.07	0.028

**Table 5:** **Enriched ingenuity pathway analysis (IPA) categories including differentially expressed genes in the depressed group**.

**IPA network top 5**	**Genes**	**IPA score**
DNA replication, recombination, and repair, cellular assembly and organization, cell cycle	*ARID4A* (*p* = 0.037); *ATRX* (*p* = 0.049); *EIF5B* (*p* = 0.021); *JMJD1C* (*p* = 0.034); *KIF23* (*p* = 0.028); *KIF18A* (*p* = 0.039); *LRP2* (*p* = 0.039); *NAP1L1* (*p* = 0.044); *NCKAP1* (*p* = 0.043); *PDE3B* (*p* = 0.040); *RAD23A* (*p* = 0.013); *SMC2* (*p* = 0.034); *SMC4* (*p* = 0.024); *SMC5* (*p* = 0.011); *SMC6* (*p* = 0.026); *TTK* (*p* = 0.049)	27
Cell cycle, cancer, connective tissue disorders	*CHD1* (*p* = 0.026); *CHD9* (*p* = 0.026); *DNAJC10* (*p* = 0.028); *FZD6* (*p* = 0.018); *HOMER1* (*p* = 0.048); *N4BP2L2* (*p* = 0.029); *NIPBL* (*p* = 0.046); *PTBP2* (*p* = 0.041); *SKIV2L2* (*p* = 0.041); *SNAPC1* (*p* = 0.046), *YEATS4* (*p* = 0.026); *ZNF638* (*p* = 0.035)	18
Cellular movement, hematological system development and function, immune cell T rafficking	*CENPE* (*p* = 0.026); *COPS2* (*p* = 0.008); *LUC7L3* (*p* = 0.026); *MPP6* (*p* = 0.034); *OTUD6B* (*p* = 0.046); *PIBF1* (*p* = 0.044); *ROCK1* (*p* = 0.023); *SENP6* (*p* = 0.021); *SENP7* (*p* = 0.023); *STXBP3* (*p* = 0.026); ZNF146 (*p* = 0.028)	16
Cardiovascular system development and function, organismal development, visual system development and function	*APOC1* (*p* = 0.034); *CHRM3* (*p* = 0.021); *DEK* (*p* = 0.039); *DNM1L* (0.048); *ERBB2IP* (0.025); *PGF* (*p* = 0.013); *PPP1R12A* (*p* = 0.021); *RALGPS2* (*p* = 0.028); *RIF1* (*p* = 0.039); *ROCK2* (*p* = 0.021)	14
Molecular transport, RNA trafficking, connective tissue disorders	*DGKH* (*p* = 0.026); *FNBP1L* (*p* = 0.046); *KTN1* (*p* = 0.021); *NKTR* (*p* = 0.046); *RNF19A* (*p* = 0.048); *THOC2* (*p* = 0.028)	9

**Table 6:** **Canonical pathway analysis of the depressed group**.

**Canonical pathway**	**Genes**	***P*-value**
Actin nucleation by ARP-WASP complex	*PPP1R12A, ROCK1, ROCK2*	0.003
RhoA signaling	*KTN1, PPP1R12A, ROCK1, ROCK 2*	0.029
VEGF signaling	*PGF, ROCK1, ROCK2*	0.011
Protein kinase A signaling	*AKAP9, ANAPC4, PDE3B, PPP1R12A, ROCK1, ROCK2*	0.011
1D-myo-inositol Hexakisphosphate Biosynthesis V (from Ins(1,3,4)P3)	*ITPK1*	0.015

### Differentially expressed genes and pathways between SSRI-treated and control placentas

Similarly, we found 109 genes to be differentially expressed between the SSRI-treated women and their respective controls at a Bonferroni-corrected significance (*P* < 0.05) threshold with a fold change of 0.5 or higher. 82 genes were down-regulated and 27 up-regulated, see Table 7. The raw microarray data is found as an excel file in the Supplementary Data. IPA analysis revealed 20 differentially expressed top genes, of which 10 were up- and 10 down-regulated (see Table 8). We then clustered placentas according to their gene expression profiles for the 20 genes that displayed differential expression (see Figure 1B). With use of IPA we focused on genes that differed in expression between placentas from antidepressant-treated women and controls. As shown in Table 9, we identified five gene networks that were significantly enriched. Of biological relevance were: (I) Infectious Disease, Cellular Assembly and Organization, Cellular Function and Maintenance with an IPA score of 13; (II) Cellular Growth and Proliferation, Inflammatory Response, Lipid Metabolism with an IPA score of 11; (III) Cell Death and Survival, Inflammatory Response, Cellular Movement with an IPA score of 9; (IV) Cell death and Survival, Liver Necrosis/Cell Death, Hematological System Development and Function with an IPA score of 8; and (V) Cardiovascular Disease, Skeletal and Muscular Disorders, Cardiovascular System Development and Function with an IPA score of 2. Further, the significant canonical pathways identified by IPA (*P* < 0.05) are shown in Table 10, along with the included genes and *p*-values. Pathways included Ephrin A Signaling, RhoA Signaling, PEDF Signaling, Breast Cancer Regulation by Stathmin1, and Signaling by Rho Family GTPases.

**Table 7:** **Significantly up and down regulated genes in the control vs. SSRI groups (− = reduction in expression levels)**.

**Gene symbol**	**Gene name**	**Probe ID**	**Log2 fold change**
*C12orf39*	chromosome 12 open reading frame 39	7954398	1.274830514
*FLJ34503*	hypothetical FLJ34503	8121569	1.266115442
*RNU4-1 /// RNU4-1B*	RNA, U4 small nuclear 1 /// RNA, U4 small nuclear 1B	7967030	0.911924177
*KRTAP19-8*	keratin associated protein 19-8	8069876	0.821169613
*OR2A7 /// OR2A4 /// LOC728377*	olfactory receptor, family 2, subfamily A, member 7 /// olfactory receptor, family 2, subfamily A, member 4 /// similar to rho guanine nucleotide exchange factor 5	8143633	0.791137617
*KRT81*	keratin 81	7963353	0.778243623
*OR2A7 /// OR2A4 /// LOC728377*	olfactory receptor, family 2, subfamily A, member 7 /// olfactory receptor, family 2, subfamily A, member 4 /// similar to rho guanine nucleotide exchange factor 5	8129558	0.774018568
*RNU4-2*	RNA, U4 small nuclear 2	7967028	0.707367007
−	−	7899484	0.693169774
*SERINC2*	serine incorporator 2	7899615	0.641601416
*APLN*	apelin	8175016	0.632933192
*ANGPTL4*	angiopoietin-like 4	8025402	0.61857093
*TUBA1C*	tubulin, alpha 1c	7955179	0.558532616
−	−	8139128	0.546472497
*S100A3*	S100 calcium binding protein A3	7920278	0.543186629
*LOC100127980*	hypothetical protein LOC100127980	8036302	0.540856558
*TECR*	trans-2,3-enoyl-CoA reductase	8101622	0.539800494
*SCARNA10*	small Cajal body-specific RNA 10	7953383	0.537535155
*RRAD*	Ras-related associated with diabetes	8001918	0.534226961
*EFNA5*	ephrin-A5	8113433	0.533194259
*CDC42EP1*	CDC42 effector protein (Rho GTPase binding) 1	8072817	0.529414661
*PCTK1*	PCTAIRE protein kinase 1	8167103	0.528119471
*SNORD116-16*	small nucleolar RNA, C/D box 116-16	7981980	0.525075622
−	−	8130181	0.511027698
−	−	7953128	0.508366155
*ORMDL3*	ORM1-like 3 (*S. cerevisiae*)	8014916	0.507840044
*ARHGEF5 /// ARHGEF5L /// LOC728377*	Rho guanine nucleotide exchange factor (GEF) 5 /// Rho guanine nucleotide exchange factor (GEF) 5-like /// similar to rho guanine nucleotide exchange factor 5	8136987	0.503949491
*MAP4K5*	mitogen-activated protein kinase kinase kinase kinase 5	7978997	−0.503225433
*FANCL /// VRK2*	Fanconi anemia, complementation group L /// vaccinia related kinase 2	8052382	−0.503353866
*AHCTF1*	AT hook containing transcription factor 1	7925622	−0.503946371
*ZNF280D*	zinc finger protein 280D	7989159	−0.505508381
*SNX6*	sorting nexin 6	7978570	−0.512758574
*CBWD3 /// CBWD5 /// CBWD6 /// LOC728877 /// CBWD7 /// LOC653510 /// CBWD2*	COBW domain containing 3 /// COBW domain containing 5 /// COBW domain containing 6 /// similar to COBW domain containing 3 /// COBW domain containing 7 /// similar to COBW domain containing 1 /// COBW domain containing 2	8155422	−0.513922351
*PHF20L1*	PHD finger protein 20-like 1	8148358	−0.514646483
*FAS*	Fas (TNF receptor superfamily, member 6)	7929032	−0.527079798
*METTL14*	methyltransferase like 14	8097066	−0.531709394
*ZNF100*	zinc finger protein 100	8035808	−0.534310441
*RGPD2 /// RGPD5 /// RGPD8 /// RGPD3 /// RGPD4 /// RGPD6 /// RGPD1 /// RANBP2*	RANBP2-like and GRIP domain containing 2 /// RANBP2-like and GRIP domain containing 5 /// RANBP2-like and GRIP domain containing 8 /// RANBP2-like and GRIP domain containing 3 /// RANBP2-like and GRIP domain containing 4 /// RANBP2-like and GRIP domain containing 6 /// RANBP2-like and GRIP domain containing 1 /// RAN binding protein 2	8044161	−0.543369485
*SHOC2*	soc-2 suppressor of clear homolog (C. elegans)	7930470	−0.550070146
*IRAK1BP1*	interleukin-1 receptor-associated kinase 1 binding protein 1	8120826	−0.550763514
*VAMP7*	vesicle-associated membrane protein 7	8171041	−0.554437451
*VAMP7*	vesicle-associated membrane protein 7	8176962	−0.554437451
*ZNF791*	zinc finger protein 791	8026007	−0.55599566
*GOLGB1*	golgin B1, golgi integral membrane protein	8089930	−0.559994665
*RGPD2 /// RGPD5 /// RGPD8 /// RGPD3 /// RGPD4 /// RGPD6 /// RGPD7 /// RGPD1 /// RANBP2*	RANBP2-like and GRIP domain containing 2 /// RANBP2-like and GRIP domain containing 5 /// RANBP2-like and GRIP domain containing 8 /// RANBP2-like and GRIP domain containing 3 /// RANBP2-like and GRIP domain containing 4 /// RANBP2-like and GRIP domain containing 6 /// RANBP2-like and GRIP domain containing 7 /// RANBP2-like and GRIP domain containing 1 /// RAN binding protein 2	8044304	−0.562605545
*FAM133B /// LOC728640 /// LOC728153*	family with sequence similarity 133, member B /// family with sequence similarity 133, member B pseudogene /// similar to FAM133B protein	8105504	−0.56577534
*STK17B*	serine/threonine kinase 17b	8057887	−0.565889797
−	−	8054532	−0.568088762
*PCM1*	pericentriolar material 1	8144812	−0.574364755
*POLK*	polymerase (DNA directed) kappa	8106303	−0.576549694
−	−	8147650	−0.578295577
*C1orf58*	chromosome 1 open reading frame 58	7909931	−0.580015227
*RGPD1 /// RGPD2 /// RGPD5 /// RGPD8 /// RGPD3 /// RGPD4 /// RGPD6 /// RGPD7 /// RANBP2*	RANBP2-like and GRIP domain containing 1 /// RANBP2-like and GRIP domain containing 2 /// RANBP2-like and GRIP domain containing 5 /// RANBP2-like and GRIP domain containing 8 /// RANBP2-like and GRIP domain containing 3 /// RANBP2-like and GRIP domain containing 4 /// RANBP2-like and GRIP domain containing 6 /// RANBP2-like and GRIP domain containing 7 /// RAN binding protein 2	8054414	−0.582592072
*RGPD2 /// RGPD5 /// RGPD8 /// RGPD3 /// RGPD4 /// RGPD6 /// RGPD7 /// RGPD1 /// RANBP2*	RANBP2-like and GRIP domain containing 2 /// RANBP2-like and GRIP domain containing 5 /// RANBP2-like and GRIP domain containing 8 /// RANBP2-like and GRIP domain containing 3 /// RANBP2-like and GRIP domain containing 4 /// RANBP2-like and GRIP domain containing 6 /// RANBP2-like and GRIP domain containing 7 /// RANBP2-like and GRIP domain containing 1 /// RAN binding protein 2	8054676	−0.586951747
*RANBP2*	RAN binding protein 2	8044263	−0.589348972
−	−	8054557	−0.592061813
*MNS1*	meiosis-specific nuclear structural 1	7989146	−0.595349364
*DNAJC13*	DnaJ (Hsp40) homolog, subfamily C, member 13	8082688	−0.601103633
*BDP1*	B double prime 1, subunit of RNA polymerase III transcription initiation factor IIIB	8106025	−0.601882298
*NKTR*	natural killer-tumor recognition sequence	8079079	−0.602491778
*ARID4A*	AT rich interactive domain 4A (RBP1-like)	7974621	−0.602522967
*CBWD3 /// CBWD5 /// CBWD6 /// CBWD7 /// LOC653510 /// CBWD2 /// CBWD1*	COBW domain containing 3 /// COBW domain containing 5 /// COBW domain containing 6 /// COBW domain containing 7 /// similar to COBW domain containing 1 /// COBW domain containing 2 /// COBW domain containing 1	8161575	−0.605839737
*BDP1*	B double prime 1, subunit of RNA polymerase III transcription initiation factor IIIB	8177560	−0.608777219
−	−	7942645	−0.611731094
*JMJD1C*	jumonji domain containing 1C	7933877	−0.612753894
*ABCC9*	ATP-binding cassette, sub-family C (CFTR/MRP), member 9	7961710	−0.613240551
*C15orf5*	chromosome 15 open reading frame 5	7990636	−0.631000244
*LAMA4*	laminin, alpha 4	8128991	−0.636829539
*FAM133B /// LOC728640*	family with sequence similarity 133, member B /// family with sequence similarity 133, member B pseudogene	8055978	−0.63846971
*PRPF40A*	PRP40 pre-mRNA processing factor 40 homolog A (*S. cerevisiae*)	8055913	−0.641694474
*ERGIC2*	ERGIC and golgi 2	7962013	−0.65241833
*ZNF644*	zinc finger protein 644	7917604	−0.664132951
*CCDC88A*	coiled-coil domain containing 88A	8052269	−0.673093507
*LYSMD3*	LysM, putative peptidoglycan-binding, domain containing 3	8113064	−0.675591155
*LRRCC1*	leucine rich repeat and coiled-coil domain containing 1	8147079	−0.676201087
*ZNF146*	zinc finger protein 146	8028186	−0.679184287
*DNTTIP2*	deoxynucleotidyltransferase, terminal, interacting protein 2	7917771	−0.687693256
−	−	8167910	−0.688561865
*GBP3 /// LOC400759 /// GBP1*	guanylate binding protein 3 /// interferon-induced guanylate-binding protein 1 pseudogene /// guanylate binding protein 1, interferon-inducible, 67kDa	7917516	−0.690433417
*CEP290*	centrosomal protein 290kDa	7965264	−0.694219538
*ABCE1*	ATP-binding cassette, sub-family E (OABP), member 1	8097647	−0.696244083
*MMRN1*	multimerin 1	8096415	−0.703832221
*CCDC55*	coiled-coil domain containing 55	8006112	−0.709824621
−	−	7989309	−0.71000087
*ZNF260*	zinc finger protein 260	8036324	−0.712626342
*KTN1*	kinectin 1 (kinesin receptor)	7974483	−0.712691264
*DNM1L*	dynamin 1-like	7954752	−0.713859601
*FER*	fer (fps/fes related) tyrosine kinase	8107208	−0.716049908
*SDCCAG1*	serologically defined colon cancer antigen 1	7978866	−0.717656644
*ZNF254*	zinc finger protein 254	8027368	−0.723859521
*ECHDC1*	enoyl Coenzyme A hydratase domain containing 1	8129379	−0.730176629
*ZNF518A*	zinc finger protein 518A	7929562	−0.730789996
*SLK*	STE20-like kinase (yeast)	7930276	−0.731081254
*RAD50*	RAD50 homolog (*S. cerevisiae*)	8107942	−0.732319601
*ROCK2*	Rho-associated, coiled-coil containing protein kinase 2	8050302	−0.73784498
*CEP170*	centrosomal protein 170kDa	7925525	−0.756625495
−	−	8047401	−0.771296325
*ANKRD12*	ankyrin repeat domain 12	8020068	−0.777177689
*ZNF721 /// ABCA11P*	zinc finger protein 721 /// ATP-binding cassette, sub-family A (ABC1), member 11 (pseudogene)	8098758	−0.777277839
*ROCK1*	Rho-associated, coiled-coil containing protein kinase 1	8022441	−0.804644966
*AKAP9*	A kinase (PRKA) anchor protein (yotiao) 9	8134122	−0.83694096
*SYCP2*	synaptonemal complex protein 2	8067305	−0.866613383
*EEA1*	early endosome antigen 1	7965436	−0.883045052
*LOC400986 /// ANKRD36B /// ANKRD36 /// LOC100289777 /// LOC100133923 /// FLJ40330*	protein immuno-reactive with anti-PTH polyclonal antibodies /// ankyrin repeat domain 36B /// ankyrin repeat domain 36 /// hypothetical protein LOC100289777 /// hypothetical protein LOC100133923 /// hypothetical LOC645784	8053801	−0.883294274
*COPS2*	COP9 constitutive photomorphogenic homolog subunit 2 (Arabidopsis)	7988605	−0.883531246
*GCC2*	GRIP and coiled-coil domain containing 2	8044236	−0.919095326
−	−	8084878	−0.91918252
*NEXN*	nexilin (F actin binding protein)	7902495	−1.000773069
−	−	8098287	−1.149966378

**Table 8:** **Significantly up- and down-regulated top molecules in the control vs. SSRI group (- = reduction in expression levels)**.

**Gene symbol**	**Gene title**	**log2 Fold change**	***P*-value**
*C12orf39*	chromosome 12 open reading frame 39	1.28	0.009
*RNU4-1*	RNA, U4 small nuclear 1	0.91	0.026
*KRT81*	keratin 81	0.78	0.007
*RNU4-2*	RNA, U4 small nuclear 2	0.71	0.043
*SERINC2*	serine incorporator 2	0.64	0.038
*APLN*	apelin	0.63	0.014
*ANGPTL4*	angiopoietin-like 4	0.62	0.047
*TUBA1C*	tubulin, alpha 1c	0.56	0.027
*S100A3*	S100 calcium binding protein A3	0.54	0.042
*TECR*	trans-2,3-enoyl-CoA reductase	0.54	0.048
*ANKRD12*	ankyrin repeat domain 12	−0.78	0.042
*ZNF721*	zinc finger protein 721	−0.78	0.033
*ROCK1*	Rho-associated, coiled-coil containing protein kinase 1	−0.80	0.041
*AKAP9*	A kinase (PRKA) anchor protein (yotiao) 9	−0.84	0.017
*SYCP2*	synaptonemal complex protein 2	−0.87	0.042
*EEA1*	early endosome antigen 1	−0.88	0.015
*ANKRD36B*	ankyrin repeat domain 36B	−0.88	0.041
*COPS2*	COP9 constitutive photomorphogenic homolog subunit 2 (Arabidopsis)	−0.88	0.007
*GCC2*	GRIP and coiled-coil domain containing 2	−0.92	0.032
*NEXN*	nexilin (F actin binding protein)	−1.00	0.044

**Table 9:** **Enriched ingenuity pathway analysis (IPA) categories including differentially expressed genes in the SSRI group**.

**IPA network top 5**	**Genes**	**IPA score**
Infectious disease, cellular assembly and organization, cellular function and maintenance	*ARHGEF5* (*p* = 0.017); *ARID4A* (*p* = 0.041); *CDK*16 (*p* = 0.023); *DNAJC13* (*p* = 0.036); *EFNA5* (*p* = 0.027); *GBP1* (*p* = 0.027); *IRAK1BP1* (*p* = 0.022); *KTN1* (*p* = 0.016); *MMRN1* (*p* = 0.045); *NKTR* (*p* = 0.017); *PRPF40A* (*p* = 0.017); *RANBP2* (*p* = 0.034); *SNX6* (*p* = 0.033)	13
Cellular growth and proliferation, inflammatory response, lipid metabolism	*AKAP9* (*p* = 0.017); *ANGPTL4* (*p* = 0.047); *APLN* (*p* = 0.014); *CDC42EP1* (*p* = 0.041); *COPS2* (*p* = 0.007); *FAS* (*p* = 0.038); *KRT81* (*p* = 0.007); *MAP4K5* (*p* = 0.040); *ROCK1* (*p* = 0.041); *TUBA1C* (*p* = 0.027); *ZNF*146 (*p* = 0.035)	11
Cell death and survival, inflammatory response, cellular movement	*ANKRD12* (*p* = 0.042); *BDP1* (*p* = 0.038); *CCDC88A* (*p* = 0.048); *DNM1L* (*p* = 0.015); *FANCL* (*p* = 0.048); *FER* (*p* = 0.042); *JMJD1C* (*p* = 0.028); *ROCK2* (*p* = 0.027); *S100A3* (*p* = 0.042)	9
Cell death and survival, liver necrosis/cell death, hematological system development and function	*ABCE1* (*p* = 0.016); *CEP170* (*p* = 0.048); *EEA1* (*p* = 0.015); *LAMA4* (*p* = 0.027); *MNS1* (*p* = 0.009); *RNU4-1* (*p* = 0.026); *RRAD* (*p* = 0.041); *STK17B* (*p* = 0.035)	8
Cardiovascular disease, skeletal and muscular disorders, cardiovascular system development and function	*NEXN* (*p* = 0.044)	2

**Table 10:** **Canonical pathway analysis of the SSRI group**.

**Canonical pathway**	**Genes**	***P*-value**
Ephrin A signaling	*EFNA5, ROCK1, ROCK2*	0.001
RhoA signaling	*CDC42EP1, KTN1,ROCK1, ROCK2*	0.002
PEDF signaling	*FAS, ROCK1, ROCK2*	0.004
Breast cancer regulation by Stathmin 1	*ARHGEF5, ROCK1, ROCK2, TUBA1C*	0.012
Signaling by Rho Family GTPases	*ARHGEF5,CDC42EP1, ROCK1, ROCK2*	0.021

### Commonly altered genes in depressed and SSRI-treated groups

Of the 108 genes that were differentially expressed between the depressed and the control cases, and the 109 genes that were differentially expressed between the SSRI-treated and the control cases, only 20 genes were overlapping. These genes are displayed in Table 11.

**Table 11:** **Genes commonly altered in depressed and SSRI groups compared with controls. (− = reduction in expression levels)**.

**Gene name**	**Gene symbol**	**Probe Set ID**	**Log2 Fold change**	
			**SSRI**	**Depressed**
jumonji domain containing 1C	*JMJD1C*	7933877	−0.612753894	−0.86686842
dynamin 1-like	*DNM1L*	7954752	−0.713859601	−0.716232722
ERGIC and golgi 2	*ERGIC2*	7962013	−0.65241833	−0.820896474
kinectin 1 (kinesin receptor)	*KTN1*	7974483	−0.712691264	−0.866627304
AT rich interactive domain 4A (RBP1-like)	*ARID4A*	7974621	−0.602522967	−0.661691075
serologically defined colon cancer antigen 1	*SDCCAG1*	7978866	−0.717656644	−0.776418378
COP9 constitutive photomorphogenic homolog subunit 2 (Arabidopsis)	*COPS2*	7988605	−0.883531246	−0.975928968
Rho-associated, coiled-coil containing protein kinase 1	*ROCK1*	8022441	−0.804644966	−1.074633032
zinc finger protein 146	*ZNF146*	8028186	−0.679184287	−0.584609444
zinc finger protein 100	*ZNF100*	8035808	−0.534310441	−0.741413733
GRIP and coiled-coil domain containing 2	*GCC2*	8044236	−0.919095326	−1.024026926
Rho-associated, coiled-coil containing protein kinase 2	*ROCK2*	8050302	−0.73784498	−0.924567683
PRP40 pre-mRNA processing factor 40 homolog A (*S. cerevisiae*)	*PRPF40A*	8055913	−0.641694474	−0.652162751
natural killer-tumor recognition sequence	*NKTR*	8079079	−0.602491778	−0.63772807
−	−	8098287	−1.149966378	−0.658215787
polymerase (DNA directed) kappa	*POLK*	8106303	−0.576549694	−0.814486061
RAD50 homolog (*S. cerevisiae*)	*RAD50*	8107942	−0.732319601	−0.827079801
LysM, putative peptidoglycan-binding, domain containing 3	*LYSMD3*	8113064	−0.675591155	−0.780972234
A kinase (PRKA) anchor protein (yotiao) 9	*AKAP9*	8134122	−0.83694096	−0.935693684
PHD finger protein 20-like 1	*PHF20L1*	8148358	−0.514646483	−0.551986952

### Validation of microarray data using qPCR analysis

For validation of the microarray results we selected seven genes that were detected in top up- or down-regulated genes, in the pathway analysis or in the canonical pathway analysis in placental tissue of both depressed and antidepressant-treated women. *ROCK1* and *ROCK2* are both involved in the actin nucleation by ARP-WASP complex, RhoA signaling, VEGF Signaling and protein kinase A signaling in the canonical pathway analysis of depressed women. Moreover they also appeared in the canonical pathway analysis of ephrin A Signaling, RhoA signaling, PEDF signaling, breast cancer regulation by stathmin 1 and signaling by Rho family GTPases of antidepressant-treated women. In addition, *ROCK1* and *GCC2* belonged to the top down-regulated genes in depressed as well as antidepressant-treated women and *ROCK2* is a top down-regulated gene in the depressed women as well. *KTN1* is involved in molecular transport, RNA trafficking, connective tissue disorders pathway of depressed women, but also in infectious disease, cellular assembly and organization, cellular function and maintenance pathway of antidepressant-treated women. Moreover, *KTN1* is involved in the RhoA signaling of the canonical pathway analysis in both depressed and antidepressant women. *DNM1L* is involved in cardiovascular system development and function, organismal development, visual system development and function of the pathway analysis in depressed women but also in cell death and survival, inflammatory response, cellular movement pathway analysis of antidepressant-treated women. *NEXN* was the top down-regulated gene in antidepressant-treated women and also turned out to be involved in cardiovascular disease, skeletal and muscular disorders, cardiovascular system development and function pathway in antidepressant-treated women. Finally, *C12orf39* was chosen as it appeared to be the top up-regulated gene in antidepressant-treated women.

**Figure 2:**
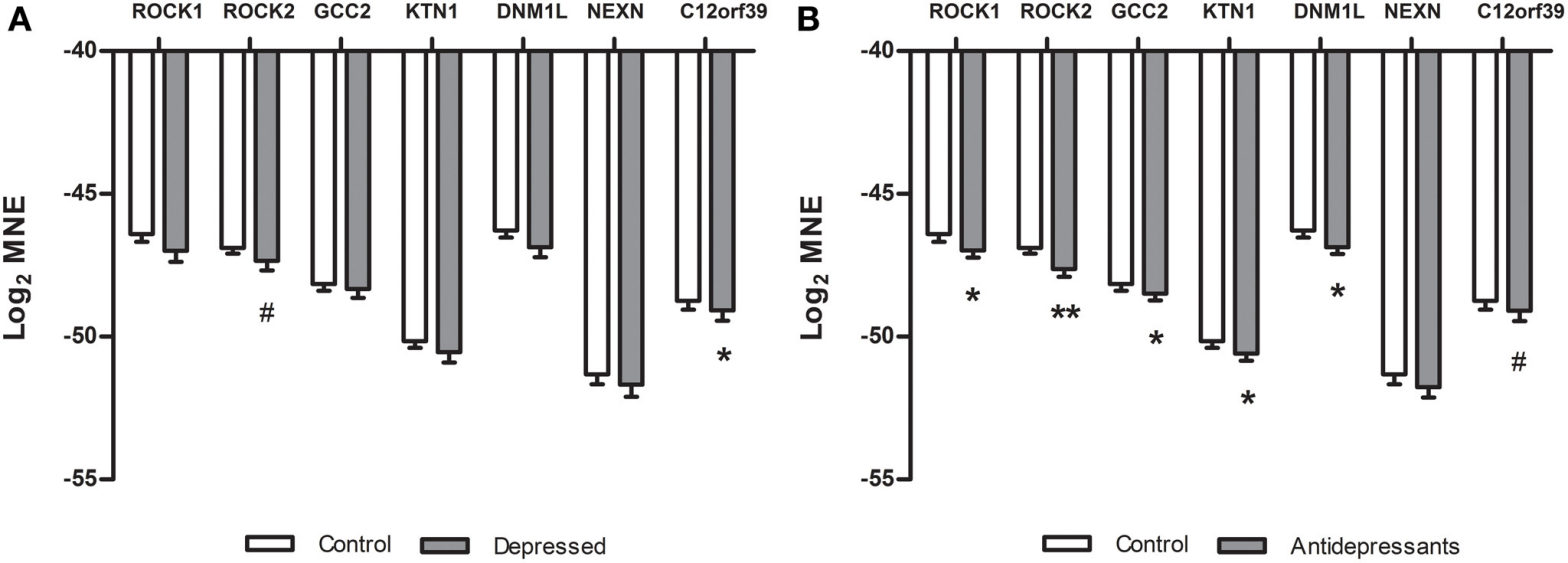
**Differences in gene expression (qPCR) between subsets in the validation study of the microarray data**. Log2 mean normalized expression (MNE) is shown for the *ROCK1, ROCK2, GCC2, KTN1, DNM1L, NEXN*, and *C12orf39* genes in depressed **(A)** and SSRI women **(B)**. **(A)** ^*^P = 0.05, ^#^*P* = 0.08, **(B)** ^*^P ≤ 0.05, ^**^P < 0.01, ^#^*P* = 0.07.

In the placenta of depressed women (Figure 2A) a significant down-regulation compared to placenta of controls was found for the *C12orf39* gene [*F*_(1, 45)_ = 3.83, *p* = 0.05] and a tendency for down-regulation of the *ROCK2* gene was found [*F*_(1, 44)_ = 3.03, *p* = 0.08]. No other genes were differentially expressed between control and depressed placentas (*NEXN* [*F*_(1, 45)_ = 0.57], *GCC2* [*F*_(1, 45)_ = 0.52], *ROCK1* [*F*_(1, 45)_ = 2.43], *DNM1L* [*F*_(1, 45)_ = 2.53] and *KTN1* [*F*_(1, 44)_ = 1.57]). When the placental gene expression was compared between antidepressant-treated women and controls (Figure 2B), we found a significant down-regulation of *ROCK1* [*F*_(1, 47)_ = 4.26, *P* < 0.05], *ROCK2* [*F*_(1, 46)_ = 9.48, *P* < 0.01], *GCC2* [*F*_(1, 47)_ = 3.78, *p* = 0.05], *KTN1* [*F*_(1, 46)_ = 6.31, *P* < 0.05], *DNM1L* [*F*_(1, 47)_ = 6.40, *P* < 0.05], and a tendency for down-regulation of *C12orf39* [*F*_(1, 47)_ = 3.39, *P* = 0.07]. The gene expression of *NEXN* [*F*_(1, 47)_ = 1.28, ns] did not differ between antidepressant-treated women and controls.

## Discussion

We performed a gene expression study in the fetal placenta of depressed women and antidepressant-treated women, and compared them with the gene expression of the placentas from women with normal pregnancies. We found that antenatal depression and antidepressant exposure during pregnancy has an influence on the gene expression of the placenta. In the microarray 108 genes were differentially expressed in women with antenatal depression, while 109 genes were differentially expressed in antidepressant-treated women. Only 20 genes were overlapping between depressed women and women on antidepressant treatment. Among the genes we chose for validation, only 2 were validated with qPCR for depressed women. In the antidepressant-treated women, 6 genes were validated, indicating a more robust effect in alterations of these genes due to antidepressant treatment during pregnancy.

Antenatal depression is a relatively heterogeneous condition with different causes (primary or secondary to somatic disease) and differential degree of endocrine disturbances. Furthermore, women with antenatal depression may also differ between the pilot microarray and the validation study as to depression severity and duration of the depressive episode. These factors may have precluded the possibility to confirm the microarray findings, and it is a major limitation that not all women in the validation part of the study were diagnosed by a structured psychiatric interview. As depression *per se* has effects on perinatal outcomes such as birth weight and gestational age (Chambers et al., 1996), suggesting that placental function is altered, further studies in more homogeneous women (and with larger sample sizes) of depression are warranted. In addition, women on antidepressant treatment during pregnancy possibly may have had a more severe depression, since continued treatment apparently was needed, and that this effect is reflected in the gene expression pattern. However, it is also possible that alterations in placental gene expression already occur because of the antenatal depression, but become (more) apparent, when antidepressants are used. Although several alterations in the gene expression of the fetal placenta were found, it remains to establish if these alterations are found in the fetus as well.

When the microarray was validated with a larger sample-size, *ROCK2* was down-regulated in both depressed and SSRI-treated women. *ROCK1* and *ROCK2* are part of the Rho-associated coiled-coil kinase family (Nakagawa et al., 1996; Amano et al., 2000) and are downstream effectors of RhoA-GTP. Rho-ROCK signaling pathways are involved in the regulation of actin cytoskeleton, cell migration and proliferation (Schofield and Bernard, 2013). In mice, *ROCK1* is highly expressed in the lung, liver, spleen, kidney and testis, whereas *ROCK2* is most abundant in the brain and heart (Nakagawa et al., 1996; Di et al., 2000; Wei et al., 2001). The role of the Rho/ROCK family in cardiovascular diseases has been extensively studied (Shi and Wei, 2013). Cardiac malformations (Diav-Citrin et al., 2008), including pulmonary hypertension (Chambers et al., 1996, 2006; Källén and Olausson, 2008; Kieler et al., 2012), have also been reported in the SSRI-exposed offspring. In the present study we found that *ROCK2* was down-regulated in antidepressant-exposed placentas (and to lesser extent in placentas of depressed women). Moreover, IPA analysis revealed that SSRI treatment affects the “Cardiovascular Disease, Skeletal and Muscular Disorders, Cardiovascular System Development and Function” network. Furthermore, it seems that the use of SSRIs intensifies the alterations in *ROCK2* expression compared to depression. Due to lowered *ROCK2* expression found in the fetal placenta of antidepressant-treated women, it is tempting to speculate that a normal expression of *ROCK2* in placenta is important for a normal function of the cardiovascular system in the fetus.

Interestingly, we also found that *NEXN* was down-regulated in antidepressant-treated women. *NEXN* is a Z-disk gene which is associated with dilated cardiomyopathy (Hassel et al., 2009). Hence, further studies are necessary to investigate the effects of down-regulated placental *NEXN* and *ROCK*2 on the development of the cardiovascular system in the fetus.

Besides the role of the Rho kinase pathway in cardiovascular diseases, a role has also been proposed for the modulation of the placental vasculature. Although expression of *ROCK1* and *ROCK2* was not different, a higher RhoA mRNA expression was found in placentae from women who suffered from preeclampsia compared with placentae from those that were normotensive (Friel et al., 2008). Interestingly, the use of antidepressants have been associated with an increased risk for preeclampsia (Palmsten et al., 2012). In addition, normal *ROCK1* and *ROCK2* activity is required for normal inner cell mass morphogenesis, which is of importance for successful fetal development (Laeno et al., 2013). These data indicate that antidepressant use, mainly SSRI, during pregnancy may influence pregnancy complications and fetal development, and that *ROCK1* and *ROCK 2* may be involved with these processes.

Another gene that was down-regulated in depressed women and to a lesser extent in antidepressant-treated women is the *C12orf39*. *C12orf39* is mainly expressed in the placenta and brain, suggesting that *C12orf39* may function in these active secretory tissues (Wan et al., 2010). With regard to its function, *C12orf39* is mainly extracellular and located in the villous trophoblasts. Trophoblasts are important in exchanges between the fetus and the mother and possess endocrine activity, releasing hormones that are important in the homoeostasis of pregnancy (Lunghi et al., 2007). In addition, trophoblasts are involved with the secretion of placental growth hormone and are related to the development of the placenta (Zeck et al., 2008). Together, these findings suggest that *C12orf39* is implicated in the regulation of placenta development by its role in the biological functions of the trophoblasts and that antenatal depression during pregnancy may influence this development. Of interest is the fact that in antidepressant-treated women the effect is no longer significant, which may indicate that antidepressants may restore the effects of the depression-induced down-regulation of *C12of39*.

*GCC2* is a peripheral membrane protein localized to the *trans*-Golgi network (Luke et al., 2003) which is involved in the maintenance of Golgi structure or transport vesicle tethering (Brown et al., 2011). More research is needed to investigate the effects of altered gene expression of *GCC2* in the placenta on the developing fetus.

Similarly, *KTN1* encodes the full kinectin which is found in the endoplasmic reticulum and is responsible for the transport of vesicles along microtubules. *KTN1* is mainly expressed in the brain, liver, ovarian, and hematopoietic cells (Tran et al., 2002; Bai et al., 2006). Of interest is that kinectin can interact with RhoA (Hotta et al., 1996), and that the RhoA signaling pathway is affected in the depressed and antidepressant-treated women. As described before normal RhoA signaling is important to prevent pregnancy complications. Possible consequences for the fetus due to the down-regulation of *KTN1* in the placenta remains to be investigated.

The last gene that was validated was *DNM1L*, which is a GTPase regulating the mitochondrial fission. In mice it was shown that ablation of the *DNM1L* gene induced defects in trophoblast giant cells and cardiomyocytes (Wakabayashi et al., 2009). Moreover, brain-specific *DNM1L* ablation caused developmental defects in the cerebellum (Wakabayashi et al., 2009). Although these results were found in knockout mice it is tempting to speculate that the down-regulation of the *DNM1L* found in the antidepressant-treated women might have an effect on embryonic and brain development as well. However, again, this effect remains to be established.

Despite the strengths of our study, such as the longitudinal nature of the study and the information on the state of the mothers mood at multiple time points, some limitations need to be discussed. First, we investigated alterations in gene expression of the fetal side of the placenta due to antenatal depression and antidepressant treatment. The results of this study may give us an indication on altered pathways in the placenta. However, the placenta is a separate organ and is not part of the fetus itself, therefore findings need to be replicated in the developing fetus. In humans this is not easily feasible therefore experiments are ongoing in a rodent study. Second, antenatal depression is a relatively heterogeneous condition and outcome of diagnoses and treatment plans (before the women entered the study) were diagnosed by different doctors which may have biased the outcome. As a result dosages and types of medication may not have been appropriate for the diagnosed depression. Nevertheless, all women did undergo the EPDS screening providing comparable data between the groups concerning the mood state at different time points during (and after) pregnancy. Third, in the validation study we included different types of antidepressants, although they were mainly SSRIs, this may have influenced the outcome of the gene expression in the SSRI treatment group.

In conclusion, gene expression in the fetal placenta is altered by antenatal depression and SSRI treatment. As more placental genes alterations were validated in a larger subset of SSRI-treated women compared to those with antenatal depression we conclude that for these subset of genes, the effects of SSRI-intake during pregnancy are more robust. It remains to be established how these differentially affected genes influence the development of the child, and whether these differences are found in the fetus as well.

### Conflict of interest statement

The authors declare that the research was conducted in the absence of any commercial or financial relationships that could be construed as a potential conflict of interest.
